# How much is enough? Effects of technical and biological replication on metabarcoding dietary analysis

**DOI:** 10.1111/mec.14779

**Published:** 2018-07-16

**Authors:** Vanessa A. Mata, Hugo Rebelo, Francisco Amorim, Gary F. McCracken, Simon Jarman, Pedro Beja

**Affiliations:** ^1^ CIBIO‐InBIO Centro de Investigação em Biodiversidade e Recursos Genéticos Universidade do Porto Vairão Portugal; ^2^ Departamento de Biologia Faculdade de Ciências Universidade do Porto Porto Portugal; ^3^ CEABN‐InBIO Centro de Ecologia Aplicada “Professor Baeta Neves” Instituto Superior de Agronomia Universidade de Lisboa Lisboa Portugal; ^4^ School of Biological Sciences University of Bristol Bristol UK; ^5^ Department of Ecology and Evolutionary Biology University of Tennessee Knoxville Tennessee; ^6^ Trace and Environmental DNA (TrEnD) Laboratory, Molecular and Life Sciences Curtin University Bentley WA Australia; ^7^ Environomics Future Science Platform CSIRO National Collections and Marine Infrastructure Crawley WA Australia

**Keywords:** bat ecology, metabarcoding, molecular diet analyses, replication, sampling design, trophic ecology

## Abstract

DNA metabarcoding is increasingly used in dietary studies to estimate diversity, composition and frequency of occurrence of prey items. However, few studies have assessed how technical and biological replication affect the accuracy of diet estimates. This study addresses these issues using the European free‐tailed bat *Tadarida teniotis*, involving high‐throughput sequencing of a small fragment of the COI gene in 15 separate faecal pellets and a 15‐pellet pool per each of 20 bats. We investigated how diet descriptors were affected by variability among (a) individuals, (b) pellets of each individual and (c) PCRs of each pellet. In addition, we investigated the impact of (d) analysing separate pellets vs. pellet pools. We found that diet diversity estimates increased steadily with the number of pellets analysed per individual, with seven pellets required to detect ~80% of prey species. Most variation in diet composition was associated with differences among individual bats, followed by pellets per individual and PCRs per pellet. The accuracy of frequency of occurrence estimates increased with the number of pellets analysed per bat, with the highest error rates recorded for prey consumed infrequently by many individuals. Pools provided poor estimates of diet diversity and frequency of occurrence, which were comparable to analysing a single pellet per individual, and consistently missed the less common prey items. Overall, our results stress that maximizing biological replication is critical in dietary metabarcoding studies and emphasize that analysing several samples per individual rather than pooled samples produce more accurate results.

## 1 INTRODUCTION

The study of animal predator diets has an old and rich history in ecology (e.g., Elton, 1927; Valverde, 1967), contributing to the understanding of species interactions, food web structure and the mechanisms driving populations and ecosystem dynamics (Layman et al., 2015; Nielsen, Clare, Hayden, Brett, & Kratina, 2017). The advent of DNA‐based molecular tools for the identification of complex multitaxa samples, that is metabarcoding, has greatly renewed the interest in dietary studies, particularly due to the high taxonomic resolution offered by this approach (e.g., De Barba et al., 2014; Kartzinel & Pringle, 2015; Lopes et al., 2015). This has been especially relevant to species whose diet is particularly difficult to study, either due to their secretive behaviour (e.g., Shehzad et al., 2012; Soininen et al., 2015) or due to difficulties to identify prey in dietary remains such as stomach contents, regurgitates and scats (e.g., Arrizabalaga‐Escudero et al., 2015; Kaunisto, Roslin, Sääksjärvi, & Vesterinen, 2017; Mollot et al., 2014). However, despite its increasingly widespread use, uncertainties and potential biases associated with the quantification of diets based on metabarcoding are still not well understood, requiring a detailed enquiry on how results are affected by different methodological options (Alberdi, Aizpurua, Gilbert, & Bohmann, 2017; Nielsen et al., 2017).

Diet studies aim to answer three main types of question about animal populations: (a) dietary diversity, generally the number of different prey species consumed; (b) dietary composition, that is the identity of the prey species consumed; and (c) the contribution of each prey species to the diet, quantified as the proportion in numbers, biomass or energetic content (e.g., Baker, Buckland, & Sheaves, 2014; Klare, Kamler, & MacDonald, 2011; Whitaker, McCracken, & Siemers, 2009). Surprisingly, there is a significant knowledge gap on the ability of metabarcoding‐based studies to provide accurate estimates of dietary descriptors, particularly under field conditions and involving species with diverse diets (Nielsen et al., 2017). Despite this paucity of quantitative studies, researchers often recognize that metabarcoding can be strongly influenced by numerous factors, which should be accounted for in dietary studies. For instance, dietary descriptors can be strongly affected by amplification bias due to unequal primer binding, which leads to systematic over‐ or underestimation of the importance of some prey types relative to others (Alberdi et al., 2017; Clarke, Soubrier, Weyrich, & Cooper, 2014). Also, “universal” barcoding markers are not necessarily good metabarcoding markers and one often has to trade taxonomic resolution for taxonomic range and vice versa (Albaina, Aguirre, Abad, Santos, & Estonba, 2016; Clarke et al., 2014; Deagle, Jarman, Coissac, Pompanon, & Taberlet, 2014), although this problem is ameliorated to some extent by recent degenerate primer versions (e.g., Alberdi et al., 2017). Taxonomic assignments of amplicon sequences are frequently limited by poor reference databases for most taxonomic groups and localities (Bohmann et al., 2011), therefore hampering data interpretation. Another problem is the imperfect correlation between the proportions of sequencing reads and biomass, making it hard to establish the contribution of each prey item to the overall diet (Deagle, Thomas, Shaffer, Trites, & Jarman, 2013; Elbrecht & Leese, 2015; Piñol, Mir, Gomez‐Polo, & Agustí, 2015). Because of this, metabarcoding studies generally quantify diet in terms of frequency of occurrence (e.g., Biffi et al., 2017; Kartzinel & Pringle, 2015; Mata et al., 2016), although this does not necessarily reflect the relative dietary intake of different prey items in terms of numbers, biomass or energy (e.g., Foster, Harmsen, & Doncaster, 2010; Greenstone et al., 2010; Sheppard et al., 2005).

An important aspect often missed in metabarcoding dietary studies is the impact of both technical and biological replication on final results. Technical replication, that is the number of extractions and PCRs carried out on each sampling unit, is important because both extractions and PCRs have a random component, and a given prey item may be missed in some replicates even if it was present in the original sample. These false negatives are expected particularly if an item's DNA is scarce or if there is a negative primer bias (Ficetola et al., 2015; Pansu et al., 2015; Willerslev et al., 2014). Biological replication, that is the number of sampling units analysed per species, including for instance the number of individuals or the number of samples per individual, is important because the number of prey species detected tends to increase with the number of samples analysed. Lack of sufficient biological replication can be detected by either rarefaction or asymptotic species richness estimators, which identify sample sizes as being too small to characterize the biodiversity in a sample (Gotelli & Colwell, 2001). Likewise, the precision of frequency of occurrence estimates is low when biological replication is low, and it varies with the prevalence of the prey items, and thus, a poor description of diet may occur at low sample sizes as a mere consequence of binomial sampling (Trites & Joy, 2005). These problems are worse when there is high variation in diet composition among individuals according for instance to gender, age or individual preferences (e.g., Mata et al., 2016; Pagani‐Núñez, Valls, & Senar, 2015; Pleguezuelos & Fahd, 2004), and there may also be intraindividual variations due for instance to temporal changes in prey availability (Burgar et al., 2014; Clare, Symondson, & Fenton, 2014b; Clare et al., 2014a).

Here, we address the impacts of technical and biological replication on the results of metabarcoding dietary analysis, focusing on the European free‐tailed bat (*Tadarida teniotis*). This species was considered suitable because previous studies (Mata et al., 2016; Rydell & Arlettaz, 1994) have shown that it is a specialist predator of moths (Lepidoptera) and thus may be less affected by problems of primer bias than species feeding on a wider range of taxonomic groups. Furthermore, moths are well represented in reference barcode databases, which reduces problems due to unidentified MOTUs. Finally, metabarcoding dietary studies have often focused on bats (e.g., Arrizabalaga‐Escudero et al., 2015; Hope et al., 2014; Razgour et al., 2011), thus making it possible to evaluate the implications of our results in the context of widely used replication options. In this study, we evaluate how variability among (a) individual bats, (b) faecal pellets of each bat and (c) PCRs of each pellet affect estimates of diet diversity and composition and on the frequency of occurrence of the prey items. Also, we tested the effects of analysing pools of samples vs. separate samples per individual, as these two variants are often used in dietary studies (e.g., *pools*: Burgar et al., 2014; Clare et al., 2014a, 2014b; Krauel, Brown, Westbrook, & McCracken, 2018; *individuals*: Hope et al., 2014; Mata et al., 2016; Vesterinen, Lilley, Laine, & Wahlberg, 2013). Our results were used to analyse the level of replication required to obtain accurate descriptions of predator diets using metabarcoding.

## 2 MATERIALS AND METHODS

### 2.1 Study design

**Figure 1:**
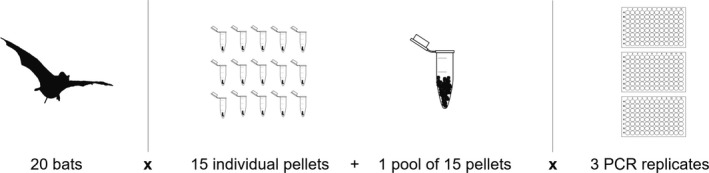
Scheme of experimental design, indicating that analyses were based on faecal pellets collected from 20 bats, with 15 separate pellets and a pool of 15 pellets per bat, and three PCR replicates per pellet/pool (*n* = 960 sampling units)

This study was based on the dietary metabarcoding analysis of 20 European free‐tailed bats (*Tadarida teniotis*), using both a 15‐pellet pool and 15 separate pellets per bat, and three PCR replicates per each pool and pellet (Figure 1). The number of individuals analysed is within or close to the range used in previous studies investigating for instance trophic structure in bird and bat assemblages (Burgar et al., 2014; Crisol‐Martínez, Moreno‐Moyano, Wormington, Brown, & Stanley, 2016; Emrich, Clare, Symondson, Koenig, & Fenton, 2014; Razgour et al., 2011; Sedlock, Krüger, & Clare, 2014). The number of pellets analysed separately for each individual is much larger than that of previous studies, which analysed either a single pellet or a pool of pellets per bat. The number of PCRs per sample is within the range (two to four) of recent studies using multiple PCRs (Biffi et al., 2017; Galan et al., 2018), although the large majority of dietary studies has been based on a single PCR per sample (e.g., Burgar et al., 2014; Crisol‐Martínez et al., 2016; Emrich et al., 2014; Razgour et al., 2011; Sedlock et al., 2014). Metabarcoding was carried out separately for each combination of bat × pellet (or pool) × PCR, yielding 960 sampling units, for which we recorded the presence/absence of each prey species. To investigate the effects of pellet sample size on the results of dietary studies, we selected randomly one PCR replicate per pellet (320 sampling units) and quantified how increasing the number of pellets analysed affected estimates of both diet diversity and the frequency of occurrence (FO) of the most important prey species. Also, we compared diet diversity and FO estimates for separate pellets and pooled samples. Finally, we used the overall sample to quantify the contribution of variation among individual bats, pellets and PCR replicates to variation in diet composition.

### 2.2 Bat pellet sampling

European free‐tailed bats (*Tadarida teniotis*) were mist‐netted at their roosts in five bridges located in northeast Portugal (N41°09′–42°00′), in April–October 2012 and 2013, under an ongoing monitoring programme (Amorim, Mata, Beja, & Rebelo, 2015). Individual bats were placed in clean cotton bags, from where guano pellets were collected. We recorded gender, age (juveniles vs. adults) and sampling date of each individual. Pellets were stored in tubes containing silica gel and refrigerated at 4°C until DNA extraction. Pellets from a subset of 143 individuals were used in a previous study to describe the diet of European free‐tailed bats (Mata et al., 2016), while for this study, we selected the pellets from a different subset of 20 individuals that had left more than 30 guano pellets in the same capture event.

### 2.3 Molecular analysis

We extracted DNA from each sample using the Stool DNA Isolation Kit (Norgen Biotek Corporation), following the manufacturer's protocol. Samples were extracted in batches of 23 plus a negative control in which no sample was added. Samples and negative controls were distributed in four 96‐well plates and kept in a freezer at −20°C until further use. DNA amplification was performed using the COI primers ZBJ‐ArtF1c and ZBJ‐ArtR2c (Zeale, Butlin, Barker, Lees, & Jones, 2011), modified to contain Illumina adaptors and a 5‐bp identification barcode. Each plate was then amplified in three independent reactions (replicates) with amplification primers containing different barcode sequences. The PCR were carried in volumes of 10 μl, comprised of 5 μl of Qiagen Multiplex PCR Master Mix, with 0.3 μl of each 10 pM primer and 1 μl of DNA extract. Cycling conditions used initial denaturing at 95°C for 15 min, followed by 35 cycles of denaturing at 95°C for 30 s, annealing at 45°C for 30 s and extension at 72°C for 30 s, with a final extension at 72°C for 10 min. Amplification success was checked by visually inspecting 2 μl of each PCR product on a 2% agarose gel. Library preparation followed the manufacturer's protocol for metagenomic sequencing (Illumina). PCR products were purified using Agencourt AMPure XP beads (Beckman Coulter) and subsequently quantified using NanoDrop and diluted to similar concentrations. All the 12 cleaned PCR plates were then pooled into a single plate, as each plate contained a different barcode. Illumina indexes were added to the cleaned PCR products using the Nextera XT Kit (Illumina), allowing individual identification of each amplified product. Indexed samples were again cleaned and then pooled at equimolar concentrations and sequenced using a whole v2 run of a MiSeq desktop sequencer (Illumina; ~0.1% coverage per sample). To test for the effect of sequencing depth on individual and pooled pellets, an additional MiSeq run was used, where one pellet and a pool were selected per individual and sequenced at “low coverage” (0.1%) and “high coverage” (1.5%). The actual coverages achieved are provided in Supporting Information Table S1.

### 2.4 Bioinformatics and prey identification

We used OBITools (Boyer et al., 2016) for general sequence processing. Briefly, paired‐end reads were aligned and assigned to samples, barcodes and primers were removed, and finally, sequences were collapsed into haplotypes. Singletons were removed, as well as sequences smaller than 155 bp and longer than 159 bp. The remaining haplotypes went through “obiclean,” a method that allows the removal of haplotypes differing 1 bp from each other, if one has a higher read count than the other in every sample. From each PCR, we further removed haplotypes representing less than 1% of the total number of reads and those containing stop codons. We then compared the haplotypes retained against known sequences within the bold database (www.boldsystems.org) and unpublished sequences of arthropods collected in northern Portugal. Haplotypes that best matched the same species were collapsed into a single taxon unit. For the haplotypes for which only family, order or class‐level identification was possible, a neighbour‐joining tree was built with all haplotypes to cluster similar sequences (>98% similarity) into distinct taxa (e.g., Cerambycidae haplotypes with divergences above 98% among them were clustered into Cerambycidae 1, Cerambycidae 2 and so on). Although this approach may artificially increase the number of taxa present in some cases, it was taken to avoid removing from further analysis taxa that are less represented on bold and for which genus or species‐level identification is often not possible.

### 2.5 Data analysis

We analysed how pellet sample size affected estimates of diet diversity by building species accumulation curves per individual, as a function of the number of pellets analysed (Colwell & Coddington, 1994). We used both the actual number of species recorded and the Chao2 estimator of species richness (Chao & Chiu, 2016). We then averaged estimates for each pellet sample size across the 20 bats analysed, to produce a mean species accumulation curve per individual. Estimates along this curve were compared to richness estimates obtained from the analysis of a pellet pool per individual. To evaluate the effects of sequencing depth, we tested for the difference in species richness in estimates based on one pellet and on a pool of 15 pellets, both at low and at high coverage. We used generalized mixed linear models (GLMM) with logit link and binomial errors, specifying individual bats as the random component, to test whether the probability of detecting a given prey item in pools was related to its frequency of occurrence in the sample of separate pellets (FOpel). Accumulation curves were carried out using the “inext” package (Hsieh, Ma, & Chao, 2016), and GLMMs were implemented using lme4 (Bates, Mächler, Bolker, & Walker, 2015).

The contribution of biological and technical replication to variation in diet composition was analysed using PerMANOVA (Anderson, 2001). Specifically, we modelled the contribution of three independent components: (a) bats, (b) pellets within bats and (c) PCRs within pellets within bats to the observed differences in species composition among sampling units. The contribution of each component while controlling for differences in degrees of freedom was estimated from the corresponding mean sum of squares (MSS). We used a nested design because we were interested in how analysing several pellets per individual contributed to variation in estimates of diet composition and not in actual dietary variation among pellets. Likewise, we were interested in the contribution of variation among PCRs of the same pellet and not in variations among PCRs per se. As a measure of the statistical significance of each component, we used an *F*‐statistic estimated with a permutation procedure (10,000 permutations), based on randomizations of the residuals of the “reduced” model (randomized residual permutation procedure—RRPP). We also used PERMANOVA to test for significant differences in prey composition inferred from pools of 15 pellets and 15 separate pellets. PerMANOVA was implemented using the function “procD.lm” of the “geomorph” package (Adams, Collyer, Kaliontzopoulou, & Sherratt, 2017).

The effects of the number of pellets analysed per individual on FO estimates of each prey species at the level of the overall sample (20 individuals) were evaluated using a simulation approach. Specifically, from each bat, we randomly sampled from *n* = 1 to 14 pellets from the overall pellet sample, to generate the empirical distribution of FO estimates at each sample size. For instance, when *n* = 2 pellets, we sampled with replacement two pellets from the pool of 15 pellets analysed per bat, for all bats, and then estimated the FO of a given prey species from the proportion of bats in which that species was detected. Repeating this procedure 10,000 times produced the empirical distribution of FO estimates for *n* = 2 pellets. We then computed the estimation error for each *n*, as the simple difference between the FO estimated using 15 pellets per bat and the FO estimated using *n* pellets per bat. To further understand the sources of variability in FO estimates, we modelled the estimation error per pellet sample size and prey species, in relation to the number of pellets analysed, the FO of that prey in the sample of 20 bats estimated using 15 pellets per individual (FOtot), the average frequency of occurrence of that prey species within individuals that consumed it (FOpel) and the first‐ and second‐order interactions between the main effects, also using 15 pellets per individual. FOtot was used to investigate whether error rates tended to be systematically lower (or higher) in prey consumed frequently by the population, whereas FOpel was used to investigate whether error rates tended to be systematically lower (or higher) in prey that was frequently consumed by particular individuals, although not necessarily at the population level. We also used beta regression to estimate whether the error rates of FO estimates in pools varied in relation to FOtot and FOpel. Simulations were implemented in the R script described in Supplementary Material, while beta regression was carried out using the “betareg” package (Cribari‐Neto & Zeileis, 2010).

## 3 RESULTS

Metabarcoding of free‐tailed bat faecal pellets detected 153 taxa from nine insect orders, of which 65.4% were Lepidoptera (Supporting Information Table S2). Most taxa (77.1%), including 95% of the Lepidoptera, were unambiguously assigned to a single species or to a group of two or three closely related species within the same genus. The seven species with the highest frequencies of occurrence (>20% of pellets) were all moths of the family Noctuidae: *Mythimna vitellina* (70.3%); *Autographa gamma* (64.3%); *Agrotis segetum* (45.3%); *Peridroma saucia* (35.7%); *Noctua pronuba/janthe* (28.7%); *Phlogophora meticulosa* (25.3%); and *Hoplodrina ambigua* (23.7%).

**Figure 2:**
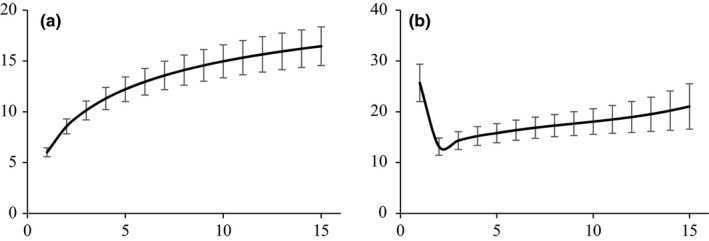
Accumulation curves for the number of (a) detected and (b) estimated (Chao2) prey species per bat, when varying the number of pellets analysed from one to 15. The curves show averages across 20 individual bats analysed, and error bars are the standard errors of mean estimates

The estimates of diet diversity per individual were strongly affected by the number of pellets analysed, either when using the actual number of species detected or when using Chao2 species richness estimator (Figure 2). On average, it was necessary to analyse seven and 12 pellets to record about 80% and 95%, respectively, of the species detected in the overall sample of 15 separate pellets per bat. Estimates of diet diversity per bat were much lower (paired‐sample *t* tests: *t* = 6.03, *df* = 19, *p* < 0.0001) in pooled samples of 15 pellets (mean ± *SD*: 5.0 ± 1.7) than in 15 pellets analysed separately (16.3 ± 8.4). Actually, either for low or for high sequencing depth, the average number of species detected was not significantly different (*low*:* t* = 4.07, *df* = 19, *p* = 0.176; *high*:* t* = 1.26, *df* = 19, *p* = 0.222) in a pool of 15 pellets (*low*: 5.3 ± 1.8; *high*: 5.4 ± 1.8) and in a single pellet (*low*: 6.3 ± 3.9; *high*: 6.2 ± 3.7). The GLMM indicated that the probability of detecting a given prey item in a pool was strongly related to its frequency of occurrence in the diet estimated from the 15 separate pellets per individual (regression coefficient [FOpel] = 5.958, *SE* = 0.6795, z = 8.768, *p *<* *0.001; Supporting Information Figure S1).

PerMANOVA showed that variation in species composition among sampling units was significantly affected by variation among individuals, pellets within individuals and PCRs within pellets (Table 1). However, the highest variation in the identity of species consumed was associated with the individual bats (MSS = 8.63). Variation associated with pellets within individuals was much lower (MSS = 0.56), but still about thirteen times higher than variation associated with PCRs within pellets (MSS = 0.04), indicating that there was little variation in the identity of species retrieved from replicate PCRs of the same pellet. PERMANOVA also showed significant differences in diet composition between the pools of 15 pellets and the 15 pellets analysed separately (*F* = 2.20, *R* ^2^ = 0.0547, *p *=* *0.003).

**Table 1:** Summary results of PerMANOVA estimating the contributions of individuals, pellets within individuals and PCRs within pellets, to overall variation in diet composition

Coefficient	*df*	SS	MS	*R* ^2^	*F*	*p*‐value
Individual	19	163.93	8.6280	0.4703	21.6758	**0.0001**
Individual:pellet	280	158.15	0.5648	0.4538	2.7493	**0.0001**
Individual:pellet:PCR	600	26.45	0.0441	0.0759	1.4981	**0.0001**
Residuals	0	0				
Total	899	348.54				

*Note*

Statistical significance was estimated from randomized residual permutation procedure, with 10,000 permutations. Significant values (*p* > 0.05) are represented in bold.

**Figure 3:**
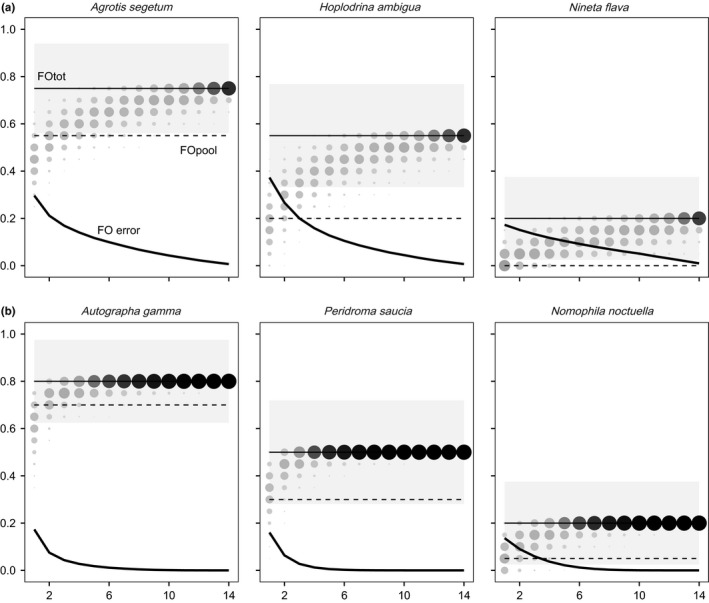
Variation in the empirical distribution of frequency of occurrence (FO) estimates (circles), in relation to the number of pellets analysed per individual, for prey items with low (a) and high (b) intraindividual FO (FOpel). Thin black lines are the FO of prey items estimated from the analysis of 15 pellets per individual (FOtot) and light shaded areas the corresponding binomial confidence interval. Thick black lines represent the mean error of FO estimates. Dashed lines represent estimated FO from pools (FOpool)

**Figure 4:**
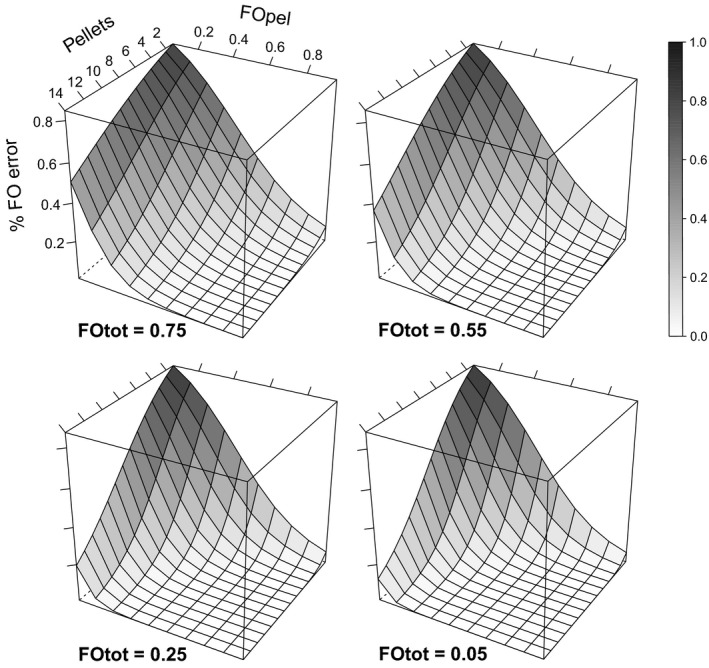
Fitted response surfaces inferred from a beta regression model showing how the error rates of frequency of occurrence (FO) estimates of prey items in the diet of European free‐tailed bats varied in relation to the number of pellets analysed and the mean frequency of occurrence of the prey items within individuals that consumed that prey (FOpel), at four levels of the frequency of occurrence of the prey items in the overall bat sample (FOtot, *n* = 20)

**Figure 5:**
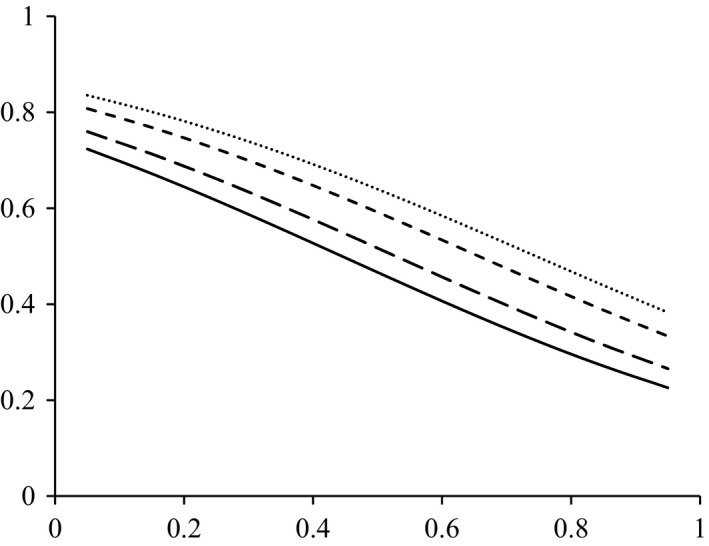
Fitted response curves inferred from a beta regression model showing how the error rates of frequency of occurrence (FO) estimates of prey items in pooled samples varied in relation to the frequency of occurrence within the pellets of individual bats (FOpel), at four levels of the frequency of occurrence of the prey items in the overall bat sample (*n* = 20; FOtot = 0.75, 0.55, 0.25 and 0.05 for black line, large dash line, small dash line and point line, respectively)

Variation in the mean FO estimates in relation to the number of pellets analysed per individual showed a consistent pattern, being strongly underestimated when the number of pellets analysed was low and progressively converging to the “true” value with increasing pellet sample size (Figure 3). Accordingly, the mean error rates of the estimates were particularly high when just one or two pellets were analysed per bat, but they declined thereafter. The beta regression model indicated that variation in the error rates of FO estimates was largely accounted for (pseudo *R* ^2^ = 0.84) by the significant effects of variation in the number of pellets analysed and the frequencies of occurrence of the prey item in the sample of 20 bats (FOtot) and in the sample of 15 pellets per bat (FOpel) (Supporting Information Table S3). The error rates always declined with the number of pellets analysed per individual, but for a given sample size, the error rates tended to be higher for species with high FOtot (i.e., prey items consumed frequently by the population) and that they tended to be lower for species that had higher FOpel (i.e., prey items consumed frequently by particular individuals) (Figure 4). The mean error rate of FO estimates was much higher (*t* = −29.35, *df* = 134, *p* < 0.0001) in pool samples of 15 pellets (82.4% ± 32.5%) than in 14 pellets analysed separately (2.2% ± 2.9%). Regarding sequencing coverage, either for low or for high sequencing depth, the error rates were similar, but significantly higher (*low*:* t* = −3.13, *df* = 134, *p* = 0.002; *high*:* t* = −2.46, *df* = 134, *p* = 0.015) in a pool of 15 pellets (*low*: 80.5% ± 34.0%; *high*: 79.5% ± 34.5%) than in a single pellet (*low*: 69.5% ± 40.7%; *high*: 71.6% ± 40.0%). Beta regression indicated that FOpel was the main factor affecting variation in the error rate of pool FO estimates across prey items (Figure 5; Supporting Information Table S4).

## 4 DISCUSSION

The results of our empirical case study focusing on the European free‐tailed bat clearly show the impact of technical and biological replication on the results of metabarcoding studies of animal predator diets. Specifically, we show strong effects of (a) the number of samples analysed per individual on estimates of diet diversity; (b) the number of individuals, samples per individual and, to a much lesser extent, the number of PCRs per sample on estimates of diet composition; and (c) the number of pellets per individual on estimates of frequency of occurrence of prey items. Also, we show that analysing pools of samples provides much poorer results than analysing separate samples to estimate diet descriptors. Therefore, our results demonstrate the importance of the levels of biological replication for adequately describing diets using metabarcoding. These results suggest that the small sample sizes in the range currently used by many studies may be insufficient to provide robust estimates of diet descriptors. However, when species are rare or otherwise difficult to sample, more limited sampling may still be useful to provide overviews of the prey consumed.

Although our results are based on a single case study that may be affected by some idiosyncrasies and limitations, this is unlikely to affect the generality of our conclusions to a significant extent. One possibility is that our results were largely driven by the particular species studied, as it consumes a wide range of different prey items (Mata et al., 2016; this study), and thus, it may require higher levels of replication than species with less diverse diets. Although diverse diets may indeed be more difficult to estimate (Nielsen et al., 2017), there are many species such as insectivore bats and birds that feed on a very wide range of taxa and thus may be as prone to insufficient biological replication as European free‐tailed bats. Another limitation is that we did not have information on the “true” diet, against which our metabarcoding results could be compared. Previous field studies have circumvented this problem by comparing metabarcoding results with those from visual or stable isotope analysis (Nielsen et al., 2017), but this is not without problems, because all methods have their own errors and biases. Therefore, these comparisons do not show which method is closer to the “truth,” but only whether different methods provide consistent results. In these circumstances, we believe that our approach of assessing how estimates of diet descriptors vary with replication levels is warranted, although further research is needed on the extent to which the method provides accurate estimates of what is actually eaten by free‐ranging animals. Finally, our study was based on the analysis of just 20 bats, with all pellets of each bat collected in the same night, and thus, it might be argued that our own study had insufficient biological replication. Although this sample size is comparable to that of previous studies, we recognize that it may be insufficient to describe in detail the diet of European free‐tailed bats. However, it highlights the difficulties of accurately estimating what 20 individuals have eaten during a single night, thereby emphasizing the challenges of inferring diets for entire populations over long time frames. This problem is not restricted to DNA metabarcoding studies of diet, however, except that their increased sensitivity of detection will make real biological variation in diet more detectable. Population diet is an inherently complicated ecological trait to characterize by any methodology, and this has been noted in the past for many dietary studies using different methodologies (Nielsen et al., 2017).

Our results support the view that technical replication affects the estimates of diet descriptors (e.g., Alberdi et al., 2017; Pansu et al., 2015; Willerslev et al., 2014), although its impact was much lower than that of biological replication. Although there was variation among PCR replicates in the composition of prey items, this was about 13 times lower than variation among pellets of the same individual bat and about 200 times lower than variation among bats. The low variation among PCR replicates suggests that prey DNA concentration was high and its degradation was low in bat faecal pellets, which are factors known to affect the amount of false positives and negatives and thus technical reproducibility in metabarcoding studies (Ficetola et al., 2015). In contrast to PCR replicates, the magnitude of variation among individuals was particularly striking, suggesting that different individuals fed on different prey items. Reasons for this are unknown, but they may be related to the effects of season, gender, age or foraging habitat. Random factors may also have played a major role, related to haphazard encounters between each foraging bat and a particular set of prey items in the night when pellets were collected. Variation among pellets of the same individual is also noteworthy, with the accuracy of diet diversity and frequency of occurrence estimates increasing markedly with the number of pellets analysed. These results seem surprising, because it might be expected that different pellets collected in the same time from a single individual would be representative of a single meal consumed in that night, thereby leading to low variability in dietary information among pellets. However, bats have an extremely rapid digestion and a high passage rate of food through the digestive tract (Staliński, 1994), and thus, differences in pellet content within individuals may reflect prey consumed at different times during the same night. As a consequence, when the number of pellets analysed per individual is low, there are many prey items missed and high error rates in frequency of occurrence estimates, particularly for prey items that are consumed by many individuals, but at low frequencies by each individual.

Pooling of samples before DNA extraction has been used to reduce processing time and costs by integrating variability among multiple samples or when individual samples were difficult to separate (Burgar et al., 2014; Clare et al., 2014a, 2014b; Jedlicka, Vo, & Almeida, 2017), but our results suggest that this strategy may lead to substantial errors in the estimation of dietary descriptors. We found that pools strongly underestimated diet diversity and the frequency of occurrence of prey items, irrespective of sequencing depth, yielding results comparable to those obtained by analysing a single pellet. Prey items consumed less frequently were consistently missed when analysing pools, and there were high error rates of FO estimates for both common and rare prey items. The reason why pools did not detect more species, even with high sequencing depth, is not entirely clear as it seems somewhat counterintuitive, because the DNA from species in individual pellets should also be present in a mix of the same pellets. However, common species in a mix will become proportionally more abundant, and rare species, which appear in low quantities in just a few pellets, will show an even smaller proportion. Therefore, the most likely explanation is that low abundance templates are not detected because of competition during PCR with proportionally more abundant templates. It is also possible that during DNA extraction, pooled samples might saturate the spin column and only the most common species get eluted. Nevertheless, the error in frequency of occurrence estimates is still slightly higher for pools even for common species. This is because pools seem to detect mostly what is highly abundant within individuals, meaning that the analysis of a single pellet is as likely to detect abundant species as is the analysis of a pool. It should be noted, however, that pooling may still be a necessary step when the initial DNA template is too low for extraction and amplification, although results need to be interpreted carefully given the errors associated with sample pooling revealed in our study.

Taken together, our results have important implications for the design of metabarcoding dietary studies, emphasizing the prominent role of biological replication to obtain robust estimates of diet diversity and composition, and the frequency of occurrence of prey items. In particular, the high variability reported here both among and within individuals points out that large numbers of individuals and sufficiently large numbers of samples per individual need to be analysed if the true diversity of the population's diet is to be recovered. Determination of sufficient levels of biological replication in general, however, will depend on the particular scientific questions being asked and the dietary characteristics of the species being studied. For instance, although in conventional studies of bat diets, it is generally agreed that 20–50 samples should be analysed for each ecological group under study (e.g., species, site, season, gender and age; Whitaker et al., 2009), this may or may not be sufficient dependent on the levels of variability within groups and the actual differences in the value of diet descriptors among groups. Larger sample sizes may thus be needed to detect differences in trophic niche between two species showing high intraspecific dietary heterogeneity due to gender, age or seasonal effects than between adult males and females of the same species on a given season, for example. On the other hand, smaller sample sizes may be more acceptable in studies aiming to provide broad descriptions of dietary patterns in diverse species communities, particularly when these include species that are rare or otherwise difficult to study than when testing specific hypothesis in community ecology requiring precise dietary estimates. Therefore, scoping studies may need to be conducted before embarking in full‐scale projects, using power analysis to estimate the levels of biological replication required to detect a given effect size at a predefined probability level (Ferry & Cailliet, 1996). When this is impractical, researchers may need to take a precautionary approach and try to maximize the number of samples analysed, which is increasingly feasible due to the ever lower costs of high‐throughput DNA sequencing.

## AUTHOR'S CONTRIBUTIONS

V.A.M., H.R., S.J. and P.B. designed the study. V.A.M. and F.A. collected the samples. V.A.M. performed the laboratory analysis. V.A.M., H.R. and P.B. analysed the data. V.A.M. and P.B. led the writing with substantial contributions from all authors.

## Data Availability

Filtered sequencing data and haplotype identification are available on Electronic Supplementary Material (ESM).
