# Supplementary material for: How much is enough? Effects of technical and biological replication on metabarcoding dietary analysis
Source: Mol Ecol. 2018 Jul 16;28(2):165–75. doi: 10.1111/mec.14779 (PMC7379978; doi:10.1111/mec.14779)
Supplement: Supplementary file 1 [file MEC-28-165-s001.docx]

Supplementary Materials for

**How much is enough? Effects of biological and technical replication on metabarcoding dietary analysis**

Vanessa A. Mata, Hugo Rebelo, Francisco Amorim, Gary McCracken, Simon Jarman, Pedro Beja

Submitted to *Molecular Ecology*

**Table S1.** Average number of reads obtained per PCR after complete bioinformatic filtering and cleaning.

| Run | Coverage | Pellet | Pool |
| --- | --- | --- | --- |
| 1 - PCR replicates | Low | 4,286 ± 95 (n=900) | 4,548 ± 372 (n=60) |
| 2 - Coverage experiment | Low | 5,462 ± 650 (n=20) | 5,400 ± 623 (n=20) |
|  | High | 90,456 ± 10,693 (n=20) | 101,533 ± 8553 (n=20) |

**Table S2.** Prey species detected in the diet of 20 European free-tailed bats (*Tadarida teniotis*) based on the metabarcoding of either 15 individual pellets or pools of 15 pellets.

| Order | Family | Species | No. Bats | No. Pellets | No. pools |
| --- | --- | --- | --- | --- | --- |
| Coleoptera | Cerambycidae | *Arhopalus ferus* | 3 | 37 | 2 |
|  |  | Cerambycidae 1 | 1 | 2 | 0 |
|  |  | Cerambycidae 2 | 3 | 11 | 0 |
|  |  | Cerambycidae 3 | 1 | 8 | 1 |
|  |  | Cerambycidae 4 | 1 | 1 | 0 |
|  |  | Cerambycidae 5 | 2 | 8 | 0 |
|  | Chrysomelidae | *Sphaeroderma rubidum* | 1 | 1 | 0 |
|  | Curculionidae | *Sitona discoideus* | 1 | 1 | 0 |
|  | Tenebrionidae | *Tenebrio molitor* | 3 | 8 | 0 |
| Diptera | Cecidomyiidae | Cecidomyiidae 1 | 1 | 1 | 0 |
|  | Chironomidae | Chironomidae 1 | 1 | 1 | 0 |
|  |  | *Chironomus* sp. 1 | 3 | 3 | 0 |
|  |  | *Cricotopus* sp. 1 | 1 | 1 | 0 |
|  | Culicidae | *Culex pipiens/quinquefasciata* | 11 | 33 | 0 |
|  |  | *Culiseta subochrea/annulata* | 3 | 4 | 0 |
|  | Limoniidae | *Limonia nubeculosa* | 1 | 1 | 0 |
|  | Scathophaga | *Scathophaga stercoraria* | 1 | 1 | 0 |
|  | Tachinidae | *Meigenia* sp. 1 | 1 | 3 | 0 |
|  | Tipulidae | *Tipula oleracea* | 6 | 28 | 2 |
|  |  | *Tipula* sp. 1 | 2 | 4 | 0 |
|  |  | *Tipula* sp. 2 | 1 | 2 | 1 |
|  |  | Tipulidae 1 | 1 | 1 | 0 |
|  | Unknown | Diptera 1 | 2 | 4 | 0 |
|  |  | Diptera 2 | 2 | 4 | 1 |
|  |  | Diptera 3 | 2 | 6 | 0 |
| Hemiptera | Pentatomidae | *Acrosternum gramineum* | 1 | 4 | 0 |
| Hymenoptera | Ichneumonidae | *Campoplex* sp. 1 | 1 | 1 | 0 |
| Lepidoptera | Coleophoridae | *Coleophora argenteonivea* | 1 | 5 | 0 |
|  | Crambidae | *Diasemiopsis ramburialis* | 1 | 3 | 0 |
|  |  | *Udea ferrugalis* | 7 | 29 | 3 |
|  |  | *Uresiphita gilvata* | 1 | 9 | 0 |
|  | Depressariidae | *Agonopterix capreolella/thapsiella* | 3 | 20 | 1 |
|  |  | *Agonopterix cnicella* | 2 | 3 | 1 |
|  |  | *Agonopterix heracliana* | 1 | 3 | 0 |
|  |  | *Agonopterix scopariella* | 7 | 44 | 1 |
|  |  | *Depressaria albipunctella* | 1 | 3 | 0 |
|  |  | *Depressaria badiella* | 1 | 4 | 0 |
|  |  | *Depressaria discipunctella* | 1 | 5 | 0 |
|  |  | *Depressaria douglasella* | 1 | 1 | 0 |
|  | Epermeniidae | *Epermenia aequidentellus* | 1 | 2 | 0 |
|  | Erebidae | *Autophila cataphanes* | 1 | 2 | 0 |
|  |  | *Eublemma ostrina* | 1 | 1 | 1 |
|  |  | *Lymantria dispar* | 1 | 1 | 0 |
|  | Gelechiidae | *Teleiopsis lindae/diffinis* | 2 | 2 | 0 |
|  | Geometridae | *Anarpia incertalis* | 1 | 4 | 0 |
|  |  | *Aplocera efformata* | 1 | 2 | 0 |
|  |  | *Aspitates ochrearia* | 1 | 1 | 0 |
|  |  | *Biston betularia* | 1 | 1 | 0 |
|  |  | *Camptogramma bilineata* | 5 | 23 | 1 |
|  |  | *Cataclysme uniformata* | 1 | 7 | 0 |
|  |  | *Cyclophora puppillaria* | 5 | 24 | 1 |
|  |  | *Eupithecia centaureata* | 1 | 1 | 0 |
|  |  | *Eupithecia pantellata* | 2 | 2 | 0 |
|  |  | *Gymnoscelis rufifasciata* | 2 | 9 | 1 |
|  |  | *Idaea cervantaria* | 1 | 7 | 0 |
|  |  | *Idaea degeneraria* | 1 | 3 | 0 |
|  |  | *Idaea rhodogrammaria* | 1 | 2 | 0 |
|  |  | *Idaea sardoniata* | 1 | 2 | 0 |
|  |  | *Orthonama obstipata* | 1 | 5 | 0 |
|  |  | *Rhodometra sacraria* | 6 | 37 | 2 |
|  |  | *Scopula marginepunctata* | 1 | 6 | 0 |
|  |  | *Stegania trimaculata* | 1 | 3 | 0 |
|  | Geometridae/Tortricidae | *Pachycnemia tibiaria/ Crocidosema plebejana* | 1 | 6 | 0 |
|  | Gracillariidae | *Parornix torquillella* | 1 | 3 | 0 |
|  | Noctuidae | *Agrotis bigramma* | 2 | 17 | 1 |
|  |  | *Agrotis ipsilon* | 5 | 21 | 1 |
|  |  | *Agrotis puta/catalaunensis* | 4 | 32 | 2 |
|  |  | *Agrotis segetum* | 15 | 136 | 11 |
|  |  | *Agrotis segetum/clavis* | 1 | 4 | 0 |
|  |  | *Agrotis segetum/ipsilon* | 1 | 1 | 0 |
|  |  | *Autographa gamma* | 17 | 193 | 15 |
|  |  | *Caradrina clavipalpis* | 1 | 9 | 0 |
|  |  | *Caradrina flavirena* | 5 | 29 | 3 |
|  |  | *Caradrina proxima* | 1 | 8 | 0 |
|  |  | *Chloantha hyperici* | 3 | 18 | 0 |
|  |  | *Cloantha hyperici* | 1 | 1 | 0 |
|  |  | *Cryphia algae* | 1 | 1 | 0 |
|  |  | *Cryphia algae/pallida* | 1 | 1 | 0 |
|  |  | *Cryphia sp. 1* | 1 | 5 | 0 |
|  |  | *Denticucullus pygmina* | 1 | 6 | 0 |
|  |  | *Euxoa temera* | 1 | 13 | 1 |
|  |  | *Hecatera dysodea* | 1 | 10 | 1 |
|  |  | *Helicoverpa armigera* | 2 | 11 | 1 |
|  |  | *Heliothis nubigera* | 1 | 5 | 0 |
|  |  | *Hoplodrina ambigua* | 11 | 71 | 5 |
|  |  | *Leucania loreyi* | 2 | 4 | 0 |
|  |  | *Leucania zeae/ Mythimna litoralis* | 1 | 7 | 1 |
|  |  | *Lophoterges millierei* | 1 | 5 | 0 |
|  |  | *Mormo maura* | 1 | 1 | 0 |
|  |  | *Mythimna albipuncta* | 7 | 35 | 3 |
|  |  | *Mythimna sicula* | 1 | 5 | 0 |
|  |  | *Mythimna vitellina* | 17 | 211 | 15 |
|  |  | *Noctua comes* | 4 | 22 | 0 |
|  |  | *Noctua fimbriata* | 1 | 2 | 0 |
|  |  | *Noctua orbona* | 4 | 26 | 0 |
|  |  | *Noctua pronuba/janthe* | 9 | 86 | 4 |
|  |  | *Noctua tirrenica* | 2 | 19 | 0 |
|  |  | *Nomophila noctuella* | 4 | 25 | 1 |
|  |  | *Nyctobrya muralis* | 1 | 5 | 0 |
|  |  | *Ochropleura leucogaster* | 2 | 9 | 1 |
|  |  | *Peridroma saucia* | 12 | 107 | 7 |
|  |  | *Phlogophora meticulosa* | 6 | 76 | 7 |
|  |  | *Rhyacia simulans* | 1 | 9 | 0 |
|  |  | *Thalpophila vitalba* | 1 | 2 | 0 |
|  |  | *Xestia agathina* | 1 | 8 | 0 |
|  |  | *Xestia kermesina* | 1 | 3 | 0 |
|  |  | *Xestia xanthographa* | 1 | 8 | 0 |
|  | Nolidae | *Nycteola columbana* | 3 | 5 | 0 |
|  |  | *Nycteola revayana* | 2 | 4 | 0 |
|  | Plutellidae | *Plutella xylostella* | 2 | 6 | 1 |
|  | Praydidae | *Prays fraxinella* | 2 | 4 | 0 |
|  |  | *Prays oleae* | 3 | 12 | 1 |
|  | Pyralidae | *Acrobasis consociella* | 1 | 6 | 0 |
|  |  | *Acrobasis obliqua* | 5 | 16 | 1 |
|  |  | *Ephestia elutella* | 2 | 3 | 0 |
|  |  | *Etiella zinckenella* | 1 | 3 | 0 |
|  |  | *Khorassania compositella* | 1 | 10 | 1 |
|  |  | *Matilella fusca* | 1 | 2 | 1 |
|  |  | *Synaphe punctalis* | 1 | 1 | 0 |
|  | Sphingidae | *Macroglossum stellatarum* | 1 | 10 | 1 |
|  | Tortricidae | *Cydia fagiglandana* | 1 | 1 | 0 |
|  |  | *Cydia pomonella* | 1 | 1 | 0 |
|  |  | Cydia sp. 1 | 1 | 1 | 0 |
|  |  | *Epagoge grotiana* | 1 | 1 | 0 |
|  | Yponomeutidae | *Zelleria oleastrella* | 1 | 3 | 0 |
|  | Ypsolophidae | *Ypsolopha ustella* | 1 | 1 | 0 |
|  | Unknown | Lepidoptera 1 | 3 | 7 | 0 |
| Mantodea | Empusidae | *Empusa pennata* | 1 | 3 | 0 |
| Neuroptera | Chrysopidae | *Chrysopa viridana* | 1 | 9 | 0 |
|  |  | *Chrysoperla lucasina/agilis/carnea/pallida* | 9 | 58 | 2 |
|  |  | Chrysopidae 1 | 3 | 9 | 0 |
|  |  | Chrysopidae 2 | 2 | 2 | 0 |
|  |  | *Cunctochrysa albolineata* | 1 | 1 | 0 |
|  |  | *Nineta flava* | 4 | 7 | 0 |
| Orthoptera | Acrididae | *Oedipoda caerulescens* | 1 | 2 | 1 |
|  | Gryllidae | *Gryllus campestris* | 1 | 1 | 0 |
|  | Tettigoniidae | *Platycleis affinis/albopunctata/intermedia* | 4 | 18 | 0 |
|  |  | *Tessellana tessellata* | 1 | 4 | 0 |
|  |  | *Tettigonia viridissima* | 1 | 3 | 0 |
| Trichoptera | Limnephilidae | *Micropterna fissa* | 3 | 8 | 0 |
|  |  | *Stenophylax nycterobius* | 1 | 2 | 0 |
|  |  | *Stenophylax vibex* | 1 | 4 | 0 |
|  |  | *Stenophylax* sp.1 | 2 | 4 | 0 |
| Unknown | Unknown | Insecta 1 | 3 | 5 | 0 |
|  |  | Insecta 2 | 1 | 3 | 0 |
|  |  | Insecta 3 | 1 | 1 | 0 |
|  |  | Insecta 4 | 1 | 1 | 0 |
|  |  | Insecta 5 | 1 | 1 | 0 |
|  |  | Insecta 6 | 1 | 2 | 0 |
|  |  | Insecta 7 | 2 | 2 | 0 |
|  |  | Insecta 8 | 1 | 1 | 0 |
|  |  | Insecta 9 | 1 | 3 | 0 |
|  |  | Insecta 10 | 1 | 1 | 0 |
| Total |  |  | 20 | 300 | 20 |

**Table S3.** Summary results of a Beta regression model (Pseudo R-squared = 0.8428) relating the error rates in the frequency of occurrence estimates of prey items in the diet of European free-tailed bats, in relation to the number of pellets analysed (pellets), the frequency of occurrence of each prey item in the sample of bats analysed (FOtot), and the frequency of occurrence of each prey item in pellets of each individual that consumed that item (FOpel).

| Coefficients | Estimate | Std. Error | z value | Pr(>\|z\|) |
| --- | --- | --- | --- | --- |
| (Intercept) | 2.5748 | 0.0354 | 72.8300 | <0.0001 |
| Pellets | -0.2660 | 0.0048 | -55.0570 | <0.0001 |
| FOtot | -0.7123 | 0.2662 | -2.6760 | 0.0075 |
| FOpel | -4.3645 | 0.1220 | -35.7890 | <0.0001 |
| Pellets:FOtot | 0.2073 | 0.0384 | 5.3990 | <0.0001 |
| Pellets:FOpel | -1.1104 | 0.0211 | -52.7020 | <0.0001 |
| Ftot:FOpel | 0.1340 | 0.5052 | 0.2650 | 0.7908 |
| Pellets:FOtot:FOpel | 1.0019 | 0.0783 | 12.8030 | <0.0001 |

**Table S4.** Summary results of a Beta regression model (Pseudo R-squared = 0.2691) relating the error rates in the frequency of occurrence estimates of prey items in the diet of European free-tailed bats, estimated through the analysis of pools of 15 pellets per individual, in relation to the frequency of occurrence of each prey item in the sample of bats analysed (FOtot), and the frequency of occurrence of each prey item in pellets of each individual that consumed that item (FOpel).

| Coefficients | Estimate | Std. Error | z value | Pr(>\|z\|) |
| --- | --- | --- | --- | --- |
| (Intercept) | 1.7906 | 0.2558 | 6.999 | <0.001 |
| FOtot | -0.9427 | 1.8910 | -0.498 | 0.618 |
| FOpel | -2.3303 | 0.7259 | -3.210 | 0.001 |
| Ftot:FOpel | -0.1420 | 3.1473 | -0.045 | 0.964 |

**Figure S1.**


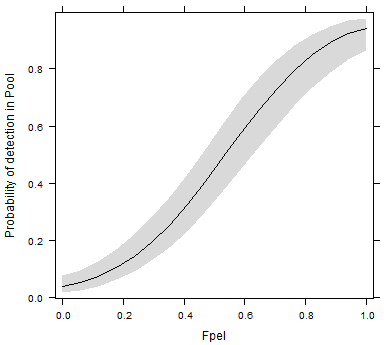


**Figure S1.** Relation between the probability of detecting a given prey item in a pool of 15 bat pellets in relation to its frequency of occurrence in an equal pellets analysed separately, as assessed from a generalized linear mixed model with logit link and binomial errors, and specifying individual bats as the random component.

**R script to permute fecal pellets**

calc.permuts <- function(presence, npell, tpell, nind, nreps) {

freq.table <- table(presence)[,2]

Freq.Total <- sum(freq.table > 0) / length(freq.table)

FreqPel <- rep(NA, nreps)

for (rep in 1:nreps) {

freq.pel <- rep(NA, nind)

for (ind in 1:nind) {

rnd.index <- sample(1:tpell, npell) + (tpell*(ind-1))

freq.pel[ind] <- (sum(presence[rnd.index, 2]) > 0) * 1

}

FreqPel[rep] <- sum(freq.pel) / length(freq.pel)

}

list(Ft = Freq.Total, Fp = FreqPel)

}

## Read data from file. Should be a table with samples as rows and species as columns. First column in the individual, while second column is the pellet.

setwd("path to working directory")

dt <- read.table("data.txt", sep="\t", header=TRUE,

stringsAsFactors=FALSE)

## Classify data to binary presences

pres <- (as.matrix(dt[,3:ncol(dt)]) > 0) * 1

## Variables for calculations (to be defined by user)

nreps <- 10000

CI <- 0.95 #for the 95% Conf Int

outdir <- "."

## Other variables automatically defined

species <- colnames(dt)[3:ncol(dt)]

inds <- unique(dt[,1])

nind <- length(inds)

total.pellets <- nrow(dt)/nind

## Prepare output result table and frequency count matrix

tl <- length(species)*(total.pellets-1)

outData <- data.frame(matrix(NA, tl, 10))

colnames(outData) <- c("Species", "Npellet", "Ftot", "Fmed",

"Fmin", "Fmax", "p.value", "CImin",

"CImax", "count")

f.size <- 0.05 # Change to get different "square" size for the freqs

f.breaks <- seq(-0.05, 1.05, f.size)

## Perform the calculations per species and per pellet

for (sp in 1:length(species)) {

f.mat <- matrix(NA, length(f.breaks)-1, total.pellets-1)

row.names(f.mat) <- paste(f.breaks[1:(length(f.breaks)-1)],

f.breaks[2:length(f.breaks)], sep="-")

## Output a pdf with histograms

pdf(file.path(outdir, paste(species[sp], ".pdf", sep="")),

paper="special", width=30/cm(1), heigh=50/cm(1))

layout(matrix(1:15, 5,3))

for (npellet in 1:(total.pellets-1)) {

presence <- data.frame(dt[,1], pres[,sp])

freqs <- calc.permuts(presence, npellet, total.pellets, nind, nreps)

Ft <- freqs$Ft

stats <- Ft - freqs$Fp

p.value <- round(sum(abs(stats) <= 0)/nreps, 4)

## Fill frequencies count matrix

f.mat[,npellet] <- table(cut(freqs$Fp, f.breaks, include.lowest=T, right=T))

## CI calculations

Fci <- Ft + c(-1,1)*qnorm(1-((1-CI)/2))*sqrt((Ft*(1-Ft))/nind)

countCI <- sum(freqs$Fp >= Fci[1] & freqs$Fp <= Fci[2])

## Histogram

sp.name <- species[sp]

title <- paste(sp.name, "\n", npellet, " pellets, ", nreps,

" replicates\nF15 = ", freqs$Ft,

", Fmed = ", round(median(freqs$Fp), 3),

", p.value = ", p.value, sep="")

hist(stats, breaks=seq(-1,1,0.05), main = title)

i <- (sp-1)*(total.pellets-1) + npellet

## Fill results table

outData[i,] <- c(species[sp], npellet, freqs$Ft, median(freqs$Fp),

min(freqs$Fp), max(freqs$Fp), p.value,

Fci[1], Fci[2], countCI)

}

dev.off()

## Save species' frequency count matrix

fMatName <- file.path(outdir, paste(species[sp], "_MAT.txt", sep=""))

write.table(f.mat, fMatName, row.names = T, col.names=T, sep=";",

quote = FALSE)

}

write.table(outData, file.path(outdir, "results.csv"), quote=FALSE,

sep='\t', row.names=FALSE, col.names=TRUE)
